# Exploring Inter-Instance Relationships within the Query Set for Robust Image Set Matching

**DOI:** 10.3390/s19225051

**Published:** 2019-11-19

**Authors:** Deyin Liu, Chengwu Liang, Zhiming Zhang, Lin Qi, Brian C. Lovell

**Affiliations:** 1 School of Information Engineering, Zhengzhou University, Zhengzhou 450001, China; iedyzzu@outlook.com (D.L.); ielqi@zzu.edu.cn (L.Q.); 2 School of Electrical and Control Engineering, Henan University of Urban Construction, Pingdingshan 467036, China; 3 School of Information Technology and Electrical Engineering, The University of Queensland, Brisbane 4072, Australia; lovell@itee.uq.edu.au; 4 School of Control Science and Engineering, Shandong University, Jinan 250100, China; zmzsdu@gmail.com

**Keywords:** image set matching, joint sparse representation, class-level sparsity, low rank regularization

## Abstract

Image set matching (ISM) has attracted increasing attention in the field of computer vision and pattern recognition. Some studies attempt to model query and gallery sets under a joint or collaborative representation framework, achieving impressive performance. However, existing models consider only the competition and collaboration among gallery sets, neglecting the inter-instance relationships within the query set which are also regarded as one important clue for ISM. In this paper, inter-instance relationships within the query set are explored for robust image set matching. Specifically, we propose to represent the query set instances jointly via a combined dictionary learned from the gallery sets. To explore the commonality and variations within the query set simultaneously to benefit the matching, both low rank and class-level sparsity constraints are imposed on the representation coefficients. Then, to deal with nonlinear data in real scenarios, the‘kernelized version is also proposed. Moreover, to tackle the gross corruptions mixed in the query set, the proposed model is extended for robust ISM. The optimization problems are solved efficiently by employing singular value thresholding and block soft thresholding operators in an alternating direction manner. Experiments on five public datasets demonstrate the effectiveness of the proposed method, comparing favorably with state-of-the-art methods.

## 1. Introduction

Image set matching (ISM) or image set classification, which regards one set of images as a sample, has recently attracted considerable attention due to its widespread applications such as video-based face recognition, multi-view object recognition, dynamic scene classification [1,2,3,4,5,6,7,8,9,10]. Compared with traditional single image based classification, ISM has the merit of incorporating information from multiple images of one set to provide all-sided description for a subject and thus achieve better matching accuracy.

Most of the existing ISM methods roughly fall into two categories, parametric and non-parametric. Parametric methods [11,12] try to model image sets with certain statistical distributions and then evaluate the similarities among those distributions. Non-parametric methods tend to assume that the image sets have underlying linear or nonlinear geometric structures and construct global set models, such as subspaces [2,13,14], manifolds [6,15,16] and affine/convex hulls [8,9,17,18,19,20,21], etc. Non-parametric methods have shown superior performance in the cases when the data distributions do not meet the models estimated by the parametric methods. Recently, some methods [1,10,22,23] have presented various further improvements following conventional non-parametric methods. Ref [22] proposes Nonlinear Subspace Feature Enhancement (NSFE) for nonlinearly embedding image sets into a space where they adhere to a more discriminative subspace structure. Ref. [23] combines multiple manifolds as the features of the original image sets, utilizing well-studied Riemannian kernels to map the original Riemannian spaces into high dimensional Hilbert spaces. In [1], aiming at alleviating the handicap that over-large affine hull usually fails in mathcing when two hulls is overlapped, Learning of Reduced Prototypes and Local Metric (LRPLM) is proposed to acquire powerful discriminative ability. Ref. [10] uses the mean vector, subspace and covariance matrix which lie on different spaces (Euclidean or manifold) to jointly represent an image set, and develops a multimodel fusion metric learning (MMFML) framework to reduce the dissimilarity between the heterogeneous spaces.

Many of the methods mentioned above, however, describe the query and gallery sets separately, and then measure the query-to-gallery distances/similarities using nearest neighbor or subspace classifiers. Firstly, they do not explore the relationships among the gallery sets which are helpful for improving ISM accuracy. Secondly, such a way of modeling separately followed by matching the models is less straightforward, or even unfair in terms of distance measurement. Finally, existing methods often depend heavily on the extracted features, i.e., different feature extraction techniques make big difference to the final matching performance. In recent years, deep learning-based image set classification methods [3,7,24,25,26] are proposed where feature extraction plays a significant role in the deep neural networks. Ref. [27] models a set of Convolutional Neural Network (CNN) features (as inputs) by a convex cone and measure the geometric similarity of convex cones for image set classification. Even some deep learning-based methods need to use extra hand-crafted features, e.g., in the method of Deep Reconstruction Models (DRM) [3], the first step of learning DRM is to compute Local Binary Pattern (LBP) features [28].

Sparse Representation has been very popular in many fields such as image classification [29,30], dictionary learning [31], color image restroation [32], recovery of remote sensing contaminated products [33], missing information reconstruction of remote-sensing images [34,35]. Sparse representation classification (SRC) [29] and Collaborative Representation Classification (CRC) [30] are two well-known classifiers which can bridge the testing sample and the training samples together under a unified framework, achieving impressive performance for single image based classification. In the unified framework, SRC or CRC implicitly describes the relationships (competition and collaboration) among the training samples while measuring directly the distances/similarities between testing and training samples. Furthermore, such methods are not sensitive to the feature extraction techniques, i.e., whatever kind of extracted feature is fed to the models based on SRC or CRC leads to good performances. Studies [36,37] either utilize the mean of the images within the query set or choose a candidate image by clustering the query set to conduct basic (single image based) SRC for image set based applications, but the basic SRC used can not exploit the rich information involved in image sets, especially in the query sets.

To accurately measure the distances between the query and gallery sets, some methods attempt to project all the sets into a collaborative or joint sparse representation framework for image set classification [19,38,39] and multimodal/multi-task recognition [40,41]. Image set based collaborative representation and classification (ISCRC) [19] describes the query set as a convex or regularized hull and represents the hull collaboratively over all the gallery sets. Group Collaborative Representation (GCR) [39] takes advantage of the relationships among gallery image sets to capture the inter-set and intra-set variations, and determines the characteristic subspaces of all the gallery sets. Methods [38,40,41] jointly represent the query set (or multiple testing features) over the gallery sets (or multiple training features) with different constraints imposed on the representation coefficients. These methods mainly explore the relationships (competition and collaboration) among different gallery sets, and even correlations and variations within gallery sets, without carefully looking into the inter-instance relationships within the query set. However, the commonality and variations within the query set are also important clues for accurate ISM tasks. In addition, existing methods are not robust to corruptions of large magnitude mixed in the query sets. Once the query sets are grossly corrupted by outliers or partial perturbations of large magnitude (e.g., occlusions), the performances of many methods will degrade drastically.

Motivated by the above insights, in this paper, we propose a new joint representation model to highlight the inter-instance relationships within the query set for improving ISM. To emphasize the inter-class discrimination among gallery sets, a class-level sparsity constraint is imposed. While for the gallery set, it is exactly the class-level sparsity constraint that implicitly connives even enlarges the variations across the instances within the query set. Therefore a constraint is imposed on the representation model by low rank regularization, to eclectically embody the commonality among instances within the query set. The combination of the two constraints explores fully both the inter-set and intra-set relationships among the query and gallery sets. The main contributions of this paper are summarized as follows:

- A joint sparse representation model with class-level sparsity constraint is chosen for ISM problem and then a low rank regularization is added to reveal thoroughly the intra-set and inter-set relationships to improve the ISM performance. To deal with nonlinear data in real scenarios, the kernelized variant of our method is presented.
- For the problem of grossly corrupted data encountered in real scenarios, which is rarely mentioned in existing ISM methods, the proposed model is extended to its robust versions to tackle different types of gross corruptions.
- The optimization challenge is solved efficiently by employing singular value thresholding and block soft thresholding operators in an alternating direction manner. The optimization algorithms for the kernelized and extended versions are modified accordingly.
- Experiments on five public datasets demonstrate that the proposed method compares favorably with competing state-of-the-art methods.

The remaining of this paper is organized as follows. Section 2.1 reviews some related work, comparing and analyzing several different joint representation models. Section 3 proposes the improved joint representation model and its kernelized version, and describes the optimization procedures for solving the objective functions. Robust extension of the proposed model and the corresponding optimization are presented in Section 4. Experimental results and analyses are showed in Section 5. Section 6 concludes this paper.

## 2. Preliminary–Related Work

### 2.1. Joint Representation

**Figure 1:**
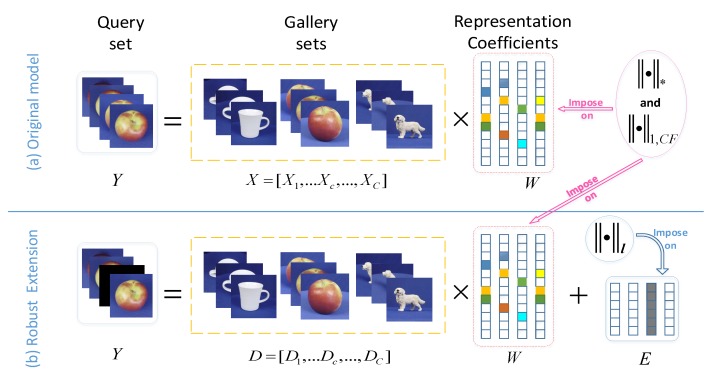
The flowchart of the proposed models: (**a**) Low Rank regularization on Class-level Sparse joint representation model (LRCS). (**b**) Extended robust model with related regularizations on the error term for different types of gross corruptions.

**Figure 2:**
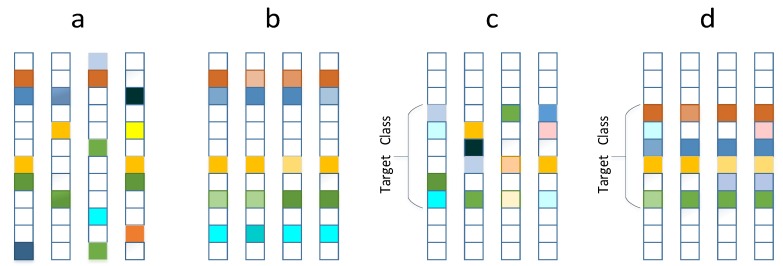
Illustration of different joint sparse representation coefficients. Each column means a representation vector and each square means a coefficient value. White squares denote zero values while colored ones denote nonzero values. (**a**) $l_{1}$-norm induced naïve joint sparsity (as in Equation (1)): simple joint sparse representation without highlighting any intra-set relationships; (**b**) Row-level sparsity (as in Equation (2)): the sparse representation vectors share a exactly the same sparsity pattern, highlighting inter-instance similarity within the query set; (**c**) Class-level sparsity (as in Equation (3)): ideally, the non-zero coefficients fall into a same class block but maybe distribute in disorder, conniving large variations among the images within the query set; (**d**) The proposed class-level sparsity with low rank regularization (as in Equation (4)): the non-zero coefficients fall into a same class block and distribute in order with slight anomaly, revealing both the commonality and the variations across images within the query set.

Joint representation with sparsity induced by $l_{1}$-norm, which is extended from SRC [29], inherits the latent discriminability resulting from the sparsity of representation coefficients. There are many images in each query set for ISM problem, hence we can consider to use this joint sparse representation to bridge query and gallery sets together. Specifically, the joint sparse representation model can represent the images within the query set via training images with joint sparsity constraint imposed on the representation coefficients. As shown in Figure 1a, given feature matrix $X=[X_{1},\ldots X_{c},\ldots ,X_{C}]$ from gallery sets of *C* classes, where $X_{c}\in R^{d\times n_{c}}$ stands for $n_{c}$ images of class c∈{1,…,C}. Each image is represented with *d* dimension feature. The feature matrix $Y\in R^{d\times n_{q}}$ of $n_{q}$ images in a query set can be represented as linear combination of the gallery set features *X*, namely Y=XW. Then the joint representation coefficient *W* can be obtained by solving the following problem:

(1) $$\hat{W}=\arg_{W}\min\frac{1}{2}{∥Y-XW∥}_{F}^{2}+\lambda {∥W∥}_{1}$$

where λ is a positive parameter, ${∥•∥}_{F}$ denotes Frobenius norm of a matrix and ${∥W∥}_{1}$ means the $l_{1}$-norm sparsity constraint that is imposed on the joint representation coefficients. This $l_{1}$-norm regularized model, whose representation coefficients are illustrated in Figure 2a, is just a trivial extension of basic sparse representation model. The naive sparsity constraint embodies latent discrimination to some degree, without reflecting clearly any intra-set or inter-set relationships. For ISM problem, inter-instance relationships within the query set is one important clue for ISM, so that more meaningful constraints should be considered to reveal real between-data relationships.

### 2.2. Joint Representation with Row-Level Sparsity

Recently, a joint representation model with row-level sparsity constraint has been proposed in the field of multimodal biometric recognition [40], which is formulated as follows:

(2) $$\hat{W}=\arg_{W}\min\frac{1}{2}{∥Y-XW∥}_{F}^{2}+\lambda {∥W∥}_{1,2}$$

where ${∥W∥}_{1,2}$ is a mixed norm defined as ${∥W∥}_{1,2}=\sum_{k=1}^{n}{∥W_{k}∥}_{2}$, and $W_{k}$ is the *K*-th row vector of *W*. The $l_{1,2}$-norm is a convex relaxation of $l_{0,2}$-norm, which applies $l_{2}$-norm on each row of and then applies $l_{0}$-norm on the resulting vector. The representation coefficients acquired by solving Equation (2) will present the row-level sparsity, as shown in Figure 2b. The nonzero rows show that the nonzero values in different coefficient column vectors share the same group of indices, meaning that every column vector of *Y* gets expressed as the linear combination of the same group of atoms (column vectors) of *X*. When applying this model for ISM, the coefficient column vectors representing different images within the query set *Y* will share the same sparsity pattern, highlighting the inter-instance similarity within the query set. However, it ignores the variations across the images within the query set, which will distort the intrinsic relationships within the query set and degrade the matching accuracy.

### 2.3. Joint Representation with Class-Level Sparsity

Similarly, another joint representation model with class-level sparsity constraint is proposed in the field of multi-task learning [41], which is formulated as follows:

(3) $$\hat{W}=\arg_{W}\min\frac{1}{2}{∥Y-XW∥}_{F}^{2}+\lambda {∥W∥}_{1,CF}$$

where ${∥W∥}_{1,CF}$ is a mixed norm defined as ${∥W∥}_{1,CF}=\sum_{c=1}^{C}{∥W_{c}∥}_{F}$, and $W_{c}\in R^{n_{c}\times n_{q}}$ denotes the components of *W* associated with the gallery features $X_{c}$ of class *c*. Similarly, the $l_{1,CF}$-norm defined here is a convex relaxation of $l_{0,CF}$-norm, ${∥\left[{∥W_{1}∥}_{F},\ldots ,{∥W_{c}∥}_{F},\ldots ,{∥W_{C}∥}_{F}\right]∥}_{0}$. The representation coefficients acquired by solving Equation (3) will present class-level sparsity, i.e., the nonzero values will group to a few classes. Ideally, as shown in Figure 2c, all the nonzero coefficients should fall into the block associated with the class *Y* belongs to. In this case, the indices of nonzero values in different coefficient column vectors locate in the same class block but could distribute in rather different positions. Hence, every column vector of *Y* gets expressed as linear combination of the same classes of but maybe different atoms of *X*. When applying this model for ISM, the class-level sparsity induced by the $l_{1,CF}$-norm constraint employs the class labels as prior supervised information. However, it maybe also connive even enlarge the variations across the images within the query set *Y*, which will impede the further performance improvement.

## 3. Low Rank Regularization on Class-Level Sparse Joint Representation Model (LRCS)

### 3.1. The Proposed LRCS Model

In terms of gallery sets, since the class-level sparsity induced by $l_{1,CF}$-norm constraint is able to employ the class label information to enhance inter-class discrimination, we prefer the class-level joint sparse characteristic for ISM task.

More importantly, the inter-instance relationships within the query set is also one important clue for ISM. In terms of query set, the $l_{1,2}$-norm constraint highlights the intra-set commonality (similarity) excessively, without considering the variations across the images within the query set. In contrast, $l_{1,CF}$-norm constraint connives large variations across the images within the query set, leading to the disorder of the distribution of non-zero coefficients located in the target class of $\hat{W}$ and thus weakening the correlations (commonality) among the images within the query set. To trade-off between the commonality and variations among the images within the query set, we consider imposing both low rank and class-level sparsity constraints on the representation coefficients for improving ISM. Based on the class-level sparse joint representation model, when the coefficients *W* is restricted to be low rank, *Y* is approximately close to XW which is also low rank, embodying the intra-set commonality and avoiding overlarge variations. Thus, Low Rank regularization on Class-level Sparse joint representation model (LRCS) is proposed, which can be formulated to solve the following minimization problem:

(4) $$\hat{W}=\arg_{W}\min\frac{1}{2}{∥Y-XW∥}_{F}^{2}+\lambda {∥W∥}_{1,CF}+\gamma {∥W∥}_{*}$$

where γ is a positive parameter controlling the low-rankness; and low rank constraint is convex relaxed into nuclear norm regularization, denoted by ${∥W∥}_{*}$ (equals the sum of singular values of matrix *W*). In real applications, the gallery set feature matrix *X* is often redundant and large, influencing the computational efficiency. Therefore, we compress feature matrix of each gallery set via dictionary learning method [31]. Finally, each class of feature matrix $X_{c}$ can be replaced with a learned class-specific dictionary $D_{c}$. By replacing *X* with $D=[D_{1},\ldots D_{c},\ldots ,D_{C}]$, Equation (4) is changed to:

(5) $$\hat{W}=\arg_{W}\min\frac{1}{2}{∥Y-DW∥}_{F}^{2}+\lambda {∥W∥}_{1,CF}+\gamma {∥W∥}_{*}$$

This model inherits the merits of unified model. Thus, not only the relationships (competition and collaboration) among gallery sets are described implicitly but also the distances between query and gallery sets can be measured directly. Furthermore, the combination of low-rankness and class-level joint sparsity constraints is exactly to trade off the commonality and variations among the query set. The representation matrix recovered via Equation (5) are class-level sparse, and the coefficients within the target class block are distributed orderly with slight anomaly, as illustrated in Figure 2d.

Once the coefficient is obtained, the class label for the whole query set is predicted according to the smallest reconstruction error:

(6) $$\hat{c}=\underset{c\in \{1,\ldots ,C\}}{\arg}\;\min residual_{c}\left(Y\right)=\underset{c\in \{1,\ldots ,C\}}{\arg}\;\min{∥Y-D_{c}{\hat{W}}_{c}∥}_{F}^{2}$$

where ${\hat{W}}_{c}$ denotes the components of the recovered $\hat{W}$ associated with the dictionary $D_{c}$.

### 3.2. The Optimization to Solve LRCS

In this section, we employ singular value thresholding and block soft thresholding operators alternately to solve the optimization problem of the proposed LRCS method. Firstly, an auxiliary variable *P* is introduced to reformulate the objective function:

(7) $$\arg_{W,P}\min\frac{1}{2}{∥Y-DW∥}_{F}^{2}+\lambda {∥P∥}_{1,CF}+\gamma {∥W∥}_{*},\;s.t.\;P=W$$

Then, the equality constraint is augmented into the objective function:

(8) $$\arg_{W,P}\min\frac{1}{2}{∥Y-DW∥}_{F}^{2}+\lambda {∥P∥}_{1,CF}+\gamma {∥W∥}_{*}+\langle T,P-W\rangle +\frac{\mu }{2}{∥P-W∥}_{F}^{2}$$

where *T* is a Lagrangian multiplier matrix, μ is a positive parameter, and $\langle A,B\rangle =tr\left(A^{T}B\right)$. Problem (8) will be solved iteratively. In each iteration, *W*, *P* and *T* will be updated alternately while keeping the other variables fixed.

(1) Update *W*: the subproblem for updating *W* has the following form

(9) $$\arg{}_{W}\min\gamma {∥W∥}_{*}+\frac{1}{2}{∥Y-DW∥}_{F}^{2}+\langle T^{k},P^{k}-W\rangle +\frac{\mu^{k}}{2}{∥P^{k}-W∥}_{F}^{2}$$

which does not have a closed-form solution. Inspired by strategy of LADMAP [42], we denote the smooth component of the above objective function (9) by $q\left(W,P^{k},T^{k}\right)=\frac{1}{2}{∥Y-DW∥}_{F}^{2}+\langle T^{k},P^{k}-W\rangle +\frac{\mu^{k}}{2}{∥P^{k}-W∥}_{F}^{2}$, then minimizing the objective function (9) can be replaced by solving the following problem:

(10) $$\arg{}_{W}\min\gamma {∥W∥}_{*}+\langle \nabla_{W}q\left(W^{k}\right),W-W^{k}\rangle +\frac{\eta^{k}}{2}{∥W-W^{k}∥}_{F}^{2}$$

where $q(W,P^{k},T^{k})$ is approximated by its linearization $\langle \nabla_{W}q\left(W^{k}\right),W-W^{k}\rangle$ at $W^{k}$ plus a proximal term $\frac{\eta^{k}}{2}{∥W-W^{k}∥}_{F}^{2}$ and $\nabla_{W}q\left(W^{k}\right)$ is the gradient of w.r.t. *W*. As long as $\eta^{k}\geq \mu^{k}+{∥D∥}_{2}^{2}$, where ${∥•∥}_{2}$ is the spectral norm of a matrix, namely, the largest singular value, the above replacement is valid. Then problem (10) has a closed-form solution given by

(11) $$W^{k+1}=\Theta_{\gamma /\eta^{k}\;}\left(W^{k}-\nabla_{W}q\left(W^{k}\right)/\eta^{k}\;\right)$$

where $\Theta_{\varepsilon }\left(A\right)=US_{\varepsilon }\left(\sum \right)V^{T}$ is the singular value thresholding operator (SVT) [43], in which $U\sum V^{T}$ is the singular value decomposition (SVD) of *A*.

(2) Update *P*: the subproblem for updating *P* has the following form

(12) $$\arg{}_{P}\min\lambda {∥P∥}_{1,CF}+\langle T^{k},P-W^{k+1}\rangle +\frac{\mu^{k}}{2}{∥P-W^{k+1}∥}_{F}^{2}$$

Involving the $l_{1,CF}$-norm constraint, problem (12) can be solved by utilizing the proximal-gradient algorithm [44]:

(13) $$P^{k+1/2\;}=W^{k+1}-\frac{T^{k}}{\mu^{k}}$$

(14) $$P_{c}^{k+1}=\max\left\{0,\left(1-\frac{\lambda /\mu^{k}\;}{{∥P_{c}^{k+1/2\;}∥}_{F}}\right)\right\}\cdot P_{c}^{k+1/2\;}$$

where $P_{c}^{k+1}$ is the *c*–th class of components of $P^{k+1}$, k+1/2 denotes an intermediate status, and Equation (14) involves a block soft thresholding operator [44].

(3) Update *T*: Finally, the Lagrange multiplier matrix is updated as follows:

(15) $$T^{k+1}=T^{k}+\mu^{k}\left(P^{k+1}-W^{k+1}\right)$$

The proposed algorithm is summarized in Algorithm 1. For faster convergence, μ can be adjusted using the adaptive updating strategy as shown in step 4 in Algorithm 1. The iteration process will terminate when the changes of the objective variables in two consecutive iterations are all below some threshold, and the difference between $P^{k+1}$ and $W^{k+1}$ reaches a preset threshold.
**Algorithm 1** Low Rank regularization on Class-level Sparse joint representation model (LRCS) and its kernelized version (K-LRCS) for Image Set Matching (ISM)**Input** **:**Learned dictionary *D*, query set feature matrix *Y* and balance parameters λ and γ. For the kernelized version, choose proper kernel and its corresponding parameters.**Output** **:**Representation coefficient matrix *W*. **Initialization:** $W^{0}=P^{0}=T^{0}=O$, $\mu^{0}=10^{-6}$, $\mu^{\max}=10^{8}$, $\rho_{0}=1.5$, $\varepsilon_{1}=\varepsilon_{2}=10^{-6}$. **Repeat** (k=0,1,…)1:Update *W* by solving (11), where, for problem (5), $\nabla_{W}q\left(W^{k}\right)=D^{T}DW^{k}-D^{T}Y-T^{k}+\mu^{k}\left(W^{k}-P^{k}\right)$ and $\eta^{k}=\mu^{k}+{∥D∥}_{2}^{2}$ for problem (17), $\nabla_{W}q\left(W^{k}\right)=\frac{1}{2}\left(K_{D,D}+{K_{D,D}}^{T}\right)W^{k}-{K_{Y,D}}^{T}-T^{k}+\mu^{k}\left(W^{k}-P^{k}\right)$ and $\eta^{k}=\mu^{k}+\frac{1}{2}\left(K_{D,D}+{K_{D,D}}^{T}\right)$2:update *P* by solving (14);3:update *T* by solving (15);4:update μ, where $\mu^{k+1}=\min\left(\mu^{\max},\rho \mu^{k}\right)$, where $\rho =\rho_{0}$ if $\max\left\{∥W^{k+1}-W^{k}∥/∥D∥\;,∥P^{k+1}-P^{k}∥/∥D∥\;\right\}\leq \varepsilon_{2}$; otherwise ρ=1; **Until** $∥W^{k+1}-P^{k+1}∥/∥D∥\;<\varepsilon_{1}$ and $\max\left\{∥W^{k+1}-W^{k}∥/∥D∥\;,∥P^{k+1}-P^{k}∥/∥D∥\;\right\}<\varepsilon_{2}$, where $\varepsilon_{2}<$

### 3.3. Computational Complexity

To analyze the computational complexity of the proposed model LRCS, we look through the procedures in the optimization algorithm. For convenience, we denote the number of the atoms of the learned combined dictionary by $n_{D}$, the number of instances within the query set by $n_{q}$, the feature dimension by *d* and the average number of iterations by *K*. We can find that at each iteration of Algorithm 1, the dominant computational costs come from the computing of gradient and singular value thresholding in Equation (11). The first two terms $D^{T}DW^{k}$ and $-D^{T}Y$ in the gradient in the step 1 in Algorithm 1 involve large computation, and $-D^{T}Y$ can be pre-computed, then the main cost for gradient estimation is $O(Kn_{q}n_{D}d+n_{q}n_{D}d)$. The cost for the singular value thresholding is $O(K{n_{D}}^{2}n_{q})$. Therefore, the computing of Equation (11) through *K* iterations requires the computation in the order of $O(Kn_{q}n_{D}d+n_{q}n_{D}d+K{n_{D}}^{2}n_{q})$. The computational overload in the steps are negligible compared to that of step 1.

### 3.4. Kernelized LRCS for Nonlinear Data

The proposed linear model will not work well when confronted with nonlinear data in real scenarios. Hence, we extend it to kernel space. The kernel function $K:R^{n}\times R^{n}$ is defined as the inner product $K\left(x_{i},x_{j}\right)=\langle \varphi \left(x_{i}\right),\varphi \left(x_{j}\right)\rangle$ where φ is an implicit mapping, projecting the vector *x* into a higher dimensional space. The projected query set features can be written as $\varphi \left(Y\right)=[\varphi \left(y_{1}\right),\varphi \left(y_{2}\right),...\varphi \left(y_{n_{t}}\right)]$ and the dictionary of gallery sets can be represented as $\varphi \left(D\right)=[\varphi \left(d_{1}\right),\varphi \left(d_{2}\right),...\varphi \left(d_{n_{D}}\right)]$, $n_{D}$ is the total number of atoms. Applying the linear joint representation in the higher dimensional space, we have φ(Y)=φ(D)W. Similar to the linear case, we seek to solve the following optimization problem:

(16) $$\hat{W}=\arg_{W}\min\frac{1}{2}{∥\varphi (Y)-\varphi (D)W∥}_{F}^{2}+\lambda {∥W∥}_{1,CF}+\gamma {∥W∥}_{*}$$

which can be reformulated w.r.t kernel matrices as

(17) $$\hat{W}=\arg{}_{W}\min\frac{1}{2}Trace\left[W^{T}K_{D,D}W-2K_{Y,D}W\right]+\lambda {∥W∥}_{1,CF}+\gamma {∥W∥}_{*}$$

where the kernel matrix $K_{A,B}$ is defined as $K_{A,B}\left(i,j\right)=\langle \varphi \left(a_{i}\right),\varphi \left(b_{j}\right)\rangle$, $a_{i}$ and $b_{i}$ are the *i*-th column of *A* and the *j*-th column of *B* respectively.

The approach to minimizing the kernelized objective function (17) has almost the same optimization procedures as in the linear case, except for the computing of the gradient of the smooth components $q(W,P^{k},T^{k})$ in the procedure (1) to update *W*. We need to replace $\frac{1}{2}\;{∥Y-DW∥}_{F}^{2}$ with $\frac{1}{2}Trace\left[W^{T}K_{D,D}W-2K_{Y,D}W\right]$ and compute the corresponding gradient.

Once $\hat{W}$ is obtained, ISM can be done by assigning the class label by

(18) $$\hat{c}=\underset{c\in \{1,\ldots ,C\}}{\arg}\;\min{∥\varphi \left(Y\right)-\varphi \left(D_{c}\right){\hat{W}}_{c}∥}_{F}^{2}\;=\underset{c\in \{1,\ldots ,C\}}{\arg}\;\min Trace\left[{{\hat{W}}_{c}}^{T}K_{D_{c},D_{c}}{\hat{W}}_{c}-2K_{Y,D_{c}}{\hat{W}}_{c}\right]$$

The kernelized version can deal with nonlinear data in ISM problem. We call Equation(17) together with Equation (18) Kernelized Low Rank regularization on Class-level Sparse joint representation model (K-LRCS). The optimization procedures are also summarized in Algorithm 2, and the complexity analysis is similar to the linear fusion.

**Algorithm 2** Robust Low Rank regularization on Class-level Sparse joint representation model (R_LRCS) for Image Set Matching (ISM)
**Input** **:**Learned dictionary *D*, query set feature matrix *Y* and balance parameters. λ, γ and β.**Output** **:**Representation coefficient matrix *W* and the corruption term *E*.**Initialization:**$W^{0}=P^{0}=T^{0}=O$, $E^{0}=O$, $\mu^{0}=10^{-6}$, $\mu^{\max}=10^{8}$, $\rho_{0}=1.5$, $\varepsilon_{1}=\varepsilon_{2}=10^{-6}$.
**Repeat** (k=0,1,…)
1:Update *W* by solving (11) where, $\nabla_{W}q\left(W^{k}\right)=D^{T}DW^{k}-D^{T}\left(Y-E^{k}\right)-T^{k}+\mu^{k}\left(W^{k}-P^{k}\right)$ and $\eta^{k}=\mu^{k}+{∥D∥}_{2}^{2}$;2:update *P* by solving (14);3:update *E* by solving (21);4:update *T* by solving (15);5:update $\mu :\mu^{k+1}=\min\left(\mu^{\max},\rho \mu^{k}\right)$ where $\rho =\rho_{0}\;\mathrm{if}\max\left\{∥W^{k+1}-W^{k}∥/∥D∥\;,∥P^{k+1}-P^{k}∥/∥D∥\;,∥E^{k+1}-E^{k}∥/∥D∥\;\right\}\leq \varepsilon_{2}$; otherwise ρ=1;
**Until**
$∥W^{k+1}-P^{k+1}∥/∥D∥\;<\varepsilon_{1}$ and $\max\left\{∥W^{k+1}-W^{k}∥/∥D∥\;,∥P^{k+1}-P^{k}∥/∥D∥\;,\right.$ $\left.∥E^{k+1}-E^{k}∥/∥D∥\;\right\}<\varepsilon_{2}$, where $\varepsilon_{2}$ >


## 4. Robust LRCS for Image Set Corruptions

When the query sets are corrupted heavily, the initially proposed model will degenerate just like most of the existing ISM methods. In this section, the proposed LRCS is extended to robust LRCS, performing against two different corruptions of large magnitude mixed in the query sets.

**Figure 3:**
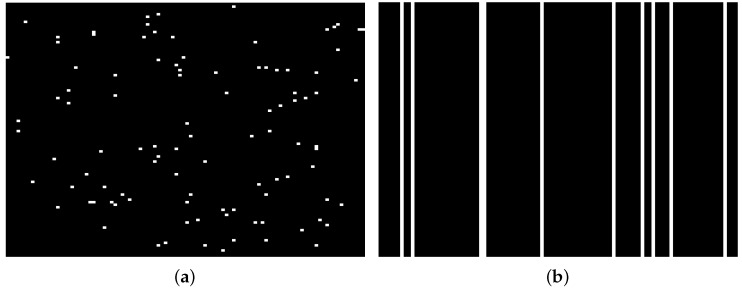
Error terms of two kinds of gross corruptions: (**a**) Random sparse corruptions. (**b**) image-specific corruption.

As shown in Figure 1b, in the case that the images within the query set are perturbed by gross corruptions, namely Y=DW+E, where *E* could be random sparse corruptions or image-specific corruptions, we should consider modifying the originally proposed model to different corruption types. As illustrated in Figure 3a, random sparse corruptions indicate that a fraction of random entries of *Y* are grossly corrupted, such as partial blurs or occlusions in some images within the query set. In other words, *E* is a spare error so that we consider adding a sparse constraint term ${∥E∥}_{0}$ to the original model. It can be convex relaxed into ${∥E∥}_{1}$. Image-specific corruptions, e.g., outliers as illustrated in Figure 3b, indicate the phenomena that a fraction of images within the query set (i.e., some columns of *Y* ) are entirely corrupted. We consider adding a column-sparse constraint term to the original model, namely ${∥E∥}_{2,0}=\#\left\{i:{∥{\left[E\right]}_{:,i}∥}_{2}\neq 0\right\}$, and convexly relax it into ${∥E∥}_{2,1}=\sum_{i}{∥{\left[E\right]}_{:,i}∥}_{2}$. Based on the previous analyses, the extended robust model is formulated as follows:

(19) $$\hat{W},\hat{E}=\arg{}_{W,E}\min\frac{1}{2}{∥Y-DW-E∥}_{F}^{2}+\lambda {∥W∥}_{1,CF}+\gamma {∥W∥}_{*}+\beta {∥E∥}_{\ell }$$

where β is a positive parameter controlling the degree of corruption, and ${∥•∥}_{\ell }$ indicates a certain regularization. The $l_{1}$-norm is used for characterizing the random sparse corruptions, and the $l_{2,1}$-norm is for dealing with image-specific corruptions.

As for the optimization to solve the problem (19), one can still employ similar procedures introduced in Section 3.2. Specifically, by introducing an auxiliary variable then we can have the augmented Lagrangian form of the objective function as follows:

(20) $$\arg{}_{W,P,E}\min\frac{1}{2}{∥Y-DW-E∥}_{F}^{2}+\lambda {∥P∥}_{1,CF}+\gamma {∥W∥}_{*}+\beta {∥E∥}_{\ell }+\langle T,P-W\rangle +\frac{\mu }{2}{∥P-W∥}_{F}^{2}$$

where the problem (20) will be solved iteratively in which each iteration will be updated alternately while keeping the other variables fixed.

(1) Update *W*: this procedure is almost the same to the procedure (1) in Section 3.2, and we just need to replace $\frac{1}{2}\;{∥Y-DW∥}_{F}^{2}$ with $\frac{1}{2}\;{∥Y-DW-E∥}_{F}^{2}$ and go on subsequent computing.
(2) Update *P*: this procedure is just the same to the procedure (2) in Section 3.2.
(3) Update *E*: the subproblem for updating *E* has the following form
  (21) $$\arg{}_{E}\min\beta {∥E∥}_{\ell }+\frac{1}{2}{∥E-(Y-DW^{k+1})∥}_{F}^{2}$$
  where if ${∥•∥}_{\ell }$ is $l_{1}$-norm, the solution to problem (21) can be computed by the soft thresholding operator $S_{\varepsilon }\left(x\right)$:
  (22) $$E^{k+1}=S_{\beta }\left(Y-DW^{k+1}\right)$$
  if ${∥•∥}_{\ell }$ is $l_{2,1}$-norm, the solution to problem (21) can be computed via:
  (23) $${\left[E^{k+1}\right]}_{:,i}=\left\{\frac{{∥{\left[Y-DW^{k+1}\right]}_{:,i}∥}_{2}-\beta }{{∥{\left[Y-DW^{k+1}\right]}_{:,i}∥}_{2}}{\left[Y-DW^{k+1}\right]}_{:,i}\;,\;\mathrm{if}\;\;{∥{\left[Y-DW^{k+1}\right]}_{:,i}∥}_{2}>\beta \right.$$
(4) Update *T*: this procedure is just the same to the procedure (3) in Section 3.2.

Once *W* and *E* are obtained, ISM can be done by assigning the class label by

(24) $$\hat{c}=\underset{c\in \{1,...,C\}}{\arg}\;\min{∥Y-D_{c}{\hat{W}}_{c}-\hat{E}∥}_{F}^{2}$$

We call Equation (19) together with Equation (24) Robust Low Rank regularization on Class-level Sparse joint representation model (R-LRCS). The proposed robust algorithm is summarized in Algorithm 2. The complexity analysis of Algorithm 2 is analog to that of Algorithm 1 as stated in Section 3.3.

## 5. Experiments

In this section, extensive experiments on five datasets are run to demonstrate the efficacy of the proposed model and its extensions. The joint representation models with different constraints are first compared showing the significance of the proposed model. Then, the comparisons with other state-of-the-art methods are presented for different ISM tasks, and finally, the impressive robustness of R-LRCS to different corruptions is verified.

### 5.1. Datasets and Preprocessing

**Honda dataset:** The Honda dataset [12] consists of 59 face video sequences which are collected from 20 different subjects. In each video sequence, there are a number of frames ranging from 12 to 645. The face images in the frames are automatically detected,cropped, and resized to 20 × 20. Finally, each video is processed to get an image set. For each class (subject), one image set is randomly selected as the gallery set for training while the rest ones are the query sets for testing.

**Figure 4:**
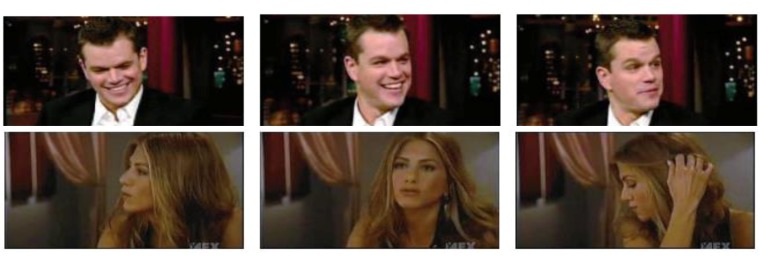
Sampling frames of two videos from YTC dataset.

**YTC dataset:** The YouTube Celebrity (YTC) dataset [45] is collected from YouTube, including 1910 videos from 47 celebrities. The face images in this dataset show large variations in expression, illumination and pose, as shown in Figure 4. Moreover, since the image compression rate is high, the resolution and quality of the images are extremely low. We extract a maximum of clearly detected face images from each video and resize them to 30 × 30 to form an image set. The dataset is equally divided into five folds, and in each fold there are nine image sets for each subject, of which three are used for training and six for testing.

**Figure 5:**
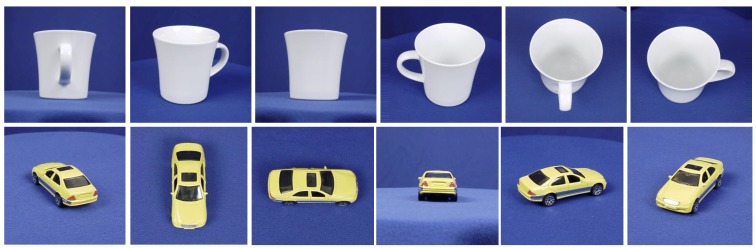
Sampling cropped images of two sets from ETH-80 dataset.

**ETH-80 dataset:** The ETH-80 dataset [46] contains eight categories of objects: cups, dogs, horses, apples, cars, cows, tomatoes and pears, as shown in Figure 5. Each category is divided into ten subcategories, each of which involves plenty of images under 41 orientations with various brands or breeds are exhibited. The images are cropped and resized to 32 × 32. Each subcategory is processed to obtain an image set and therefore there are 80 sets in total. For each object, half of the sets are randomly selected for training and the remaining are used for testing.

**UCSD Traffic dataset:** The UCSD Traffic dataset [47] contains 254 video sequences of highway traffic with varying patterns (i.e., light, medium and heavy) in various weather conditions (e.g., cloudy, raining, sunny). HoG features [48] are used to describe each frame. The experiments are performed using the splits provided with the dataset [47].

**Maryland dataset:** The Maryland dataset [49] contains 13 different classes of dynamic scenes with ten videos per class, such as avalanches and tornados. We use the last convolutional layer of the Convolutional Neural Network (CNN) model [50] as the frame descriptors. Then the dimensionality of the CNN output is reduced into 400 using Principal Component Analysis (PCA). We randomly choose seven image sets (videos) of each class for training and the remaining three for testing.

These datasets cover different sub-tasks of image set matching. The first two are for face identification, ETH-80 is for object categorization, and the last two are for scene classification. For all the datasets, the color images are converted to gray scale levels. In order to examine the anti-interference of various methods to illumination variations, histogram equalization is not used in the preprocessing stage for all the datasets. For Honda, YTC and ETH-80 datasets, we use the raw pixel values directly as features while employing different extracted features for the rest. All the features are normalized into vectors of unit one except for the query set features in the corruption experiments.

### 5.2. Experiment Setup

As for the experimental settings of the proposed method, we first compress each gallery set using a dictionary learning method such as KSVD [31] to a sub-dictionary with atoms of fixed number and stack them up to form a combined dictionary. The number is set as 50,10,20,5 on Honda, YTC, ML, Traffic datasets respectively. The gallery sets of ETH-80 are stacked up directly as a dictionary without compressing, as their sizes are already small. The balance parameters λ, γ, β are often chosen among three different orders of magnitude $\left\{0.1,0.01,0.001\right\}$. Detailed analyses on the sensitivity of parameters are described in Section 5.3. For the kernelized version of experiments, we choose the polynomial kernel.

We compare the proposed LRCS and its kernelized extension K_LRCS with some representative ISM methods, including several methods aiming at data nonlinearity, such as kernelized versions of several existing linear models and deep learning-based DRM [3]. Their abbreviations and names are shown in Table 1. For all the methods, their parameters are tuned for the best performances.

Specifically, for the MSM and linear AHISD, the thresholds for determining the subspace size are both set as 0.98. For the KAHISD, we set the bounds as −L=U=τ, where the value of τ is chosen to be 5. The upper bounds of the CHISD and KCHISD are set to U=1. For the SANP, the same weighing parameters as in [9] are taken to implement convex optimization. For the RHISCRC, we choose the $l_{1}$-norm regularized hull, and adopt the same parameter setting as in [19] for the RHISCRC and KCHISCRC. For the NN_H and NN_J, the bandwidth parameter of the KDE kernels are chosen among $\left\{0.01,0.1,1.0,10\right\}$ for different datasets. For the PDL, GCR, DRM and KRCHISD, we have the same parameter setting as in their originally proposed papers respectively. For all the kernelized methods, we use the Gaussian or polynomial kernels.

Ten-fold cross validation experiments are run for each kind of test, except on YTC dataset (five-fold) and Traffic dataset (four-fold).

### 5.3. Sensitivity of the Parameters

There are two positive parameters controlling the balance among different terms in the objective functions of the proposed model and its kernelized extension. λ controls the class-level sparsity while γ decides the low-rankness. Classification results of LRCS and K-LRCS with different parameter settings of λ and γ on the Traffic dataset are provided in Table 2 and Table 3 respectively.

The kernelized version shows more stable average recognition rates than the LRCS model, but in general, both their recognition performances do not change much. When fixing a parameter and fine tuning the other, the best performance often occurs when the two parameters are in the same order of magnitude. This implies that both of the class-level sparsity and low-rankness are at work, and they are complementary.

### 5.4. Comparisons among the Different Joint Representation Models

To compare the performances of various aforementioned joint representation models, we choose Maryland dataset as a representative to run experiments, due to the abundance of its data quantity and diversity covering many potential data structures. For convenience, we denote these models by some abbreviations as shown in Table 1. We also add a low rank regularization to a row-level sparse joint representation model, denoted by LR+L12, to participate in the comparisons.

Table 4 shows the recognition rates of various joint representation models with 10 times of testing on Maryland dataset. Form the table, it is observed that L1 has an ordinary performance since it does not highlight any relationship within the query sets. CS enhances the inter-class discrimination utilizing class label prior, so it is supposed to perform always better than L12. At test no. 1,4,5 and 10, however, the recognition rates of CS are not higher (even lower) than that of L12. The reason is that the non-zero coefficients recovered by CS distribute rather chaotically in the potential targeted class (as shown in Figure 2c, which connives even enlarges the variations across the images within the query set so that the inherent commonality (similarity) among the images is weakened. In fact, in terms of query set, L12 highlights similarity, implying extremely low rank, while probably CS has higher rank than the truth, due to the disordered distribution of the non-zero coefficients. In contrast to that, the proposed LRCS explores low-rankness with the class-level sparsity constraints. Realizing not only enhanced inter-class discrimination by class-level sparsity constraint, but also intra-set trade-off between commonality and variations by adding a low rank regularization, the proposed LRCS model performs better for ISM.

Obviously, the performance of the LR+L12 model is inferior to that of LRCS, and it is also not better (even worse) than that of L12. The reason is that the row-level sparsity has already means extreme low-rankness. Adding a low rank regularization leads the coefficients to be far from the truth, bringing worse performance. In addition, there is no significant increase in the recognition rates of K_LRCS over that of LRCS except for the smaller standard deviation meaning higher stability. Probably because the CNN features in this dataset are relatively linearly tractable and thus the linear model LRCS has already been able to present competitive matching performance.

### 5.5. Comparisons with the State-of-the-Art Methods

As shown in Table 5, we compare the proposed method with the state-of-the-art in terms of ISM performance (average recognition rates and standard deviations) on the five benchmark datasets. For average recognition rates, both linear and nonlinear versions of the proposed model show the best or near the best performance on all the datasets in comparison to their respective categories of methods. Furthermore, the standard deviations of our proposed models are relatively small on each dataset, indicating the large stability of image set matching.

The KCHISCRC [19] method performs almost the second best. Just like the proposed models, it also belongs to the category of unified modelling which models query and gallery sets together and measures their distances directly. To some extent, this demonstrates that the superiority of unified modelling over other methods modelling separately each set. Another unified modelling method RHISCRC [19] also performs well on most datasets but unsatisfactorily on the rest, especially on Traffic. The reason is that the $l_{1}$-norm constraints used both *a* and β [19] assume implicitly that only a few extremely similar images from the query and gallery sets participate in constructing hulls and modelling, where inter-class ambiguities easily cause misclassifications. Notice that sometimes the average recognition rates of K_LRCS has little advantage over (even is inferior to) that of LRCS, just as KAHISD vs. AHISD, and KCHISD vs CHISD. It can be inferred that whether the kernelization operations can help significant improvement of performance also relies on how much nonlinearity is embedded in the features used.

We also observe that many other methods perform well on some datasets while poor on the others. That is to say, their performances are not stable, depending on the datasets. For example, RT-LSVM [4] almost fails on the Maryland dataset. A probable explanation is that, as analyzed in [4], this method has rigorous limits to the relationship among the three: the number of class labels, the number of images within the query set, and the number of training images of each class, which Maryland cannot satisfy. In addition, the deep learning-based DRM method does not necessarily outperform other methods especially on the YTC and ETH-80 datasets. For the DRM method, the first step of its model is to extract the LBP features, but for fair comparisons, by removing the feature extraction step, we use the pixel values directly just as other methods on Honda, YTC, and ETH-80 datasets. Since only extracted features rather than LBP are provided on the Maryland and Traffic datasets, DRM code is not run on them. Maybe the mandatory remove of the LBP feature extraction procedure impairs the performance of DRM (the dependence of different methods on features will be further demonstrated in Section 5.6). Moreover, the small size of datasets limits the advantages of deep learning. The good news is, our proposed method shows impressive ability of dealing with nonlinearity, which is comparable to the deep model.

In terms of running time, for a normal image set, the average running time of our method varies on different datasets due to their different complexity (data quantity in every set, feature dimension, etc). For example, for LRCS, it is 1.68 s on Honda dataset while 3.38 s on YTC dataset on a PC with Intel(R) Core(TM) i7-3700 CPU and RAM 8GB. Such a running time is common compared to other methods for ISM problem [21].

### 5.6. Dependence on the Features

**Figure 6:**
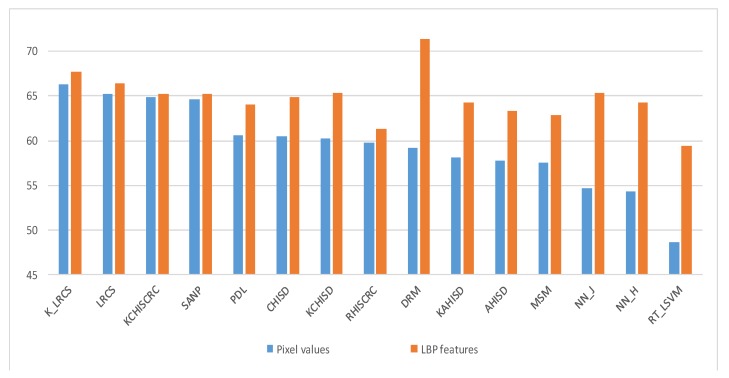
Recognition performance of different methods using two different features on YTC dataset.

To investigate the dependence of different methods on features (or feature extraction techniques), we evaluate their recognition performance on YTC dataset using two kinds of features: Pixel values and LBP features. The results are shown in Figure 6. For the convenience of observation, various methods are sorted according to the recognition rates using pixel feature from the largest to the smallest. Obviously, the proposed models perform well consistently using whatever types of features, and the performance gaps between different features are small. Conversely, most of the methods compared depend heavily on specific kind of features, indicating that it is necessary to choose suitable feature extraction techniques for better performances. For example the DRM method even takes the LBP feature extraction as the first step of its model, the recognition performance drops dramatically once removing or replacing the LBP feature.

### 5.7. Robustness Comparisons

In this section, experimental results on the Honda and ETH-80 datasets are presented to investigate the robustness of different methods to random sparse corruptions and image-specific corruptions, respectively.

**Figure 7:**
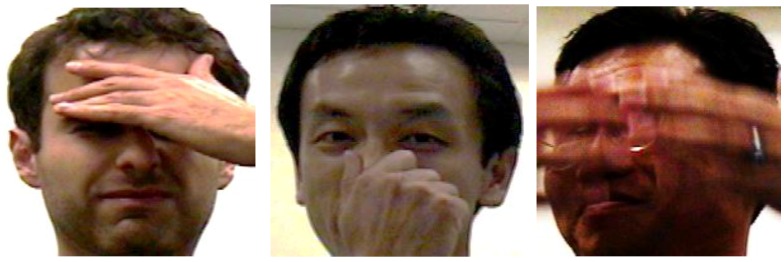
Three example frames drawn from three corrupted videos of Honda dataset with different illuminations.

To test the robustness of different methods to random partial occlusions, we choose the Honda dataset for performance evaluation. Three occluded frames from three testing videos with partial occlusion are shown in Figure 7. Since the number of the occluded videos is small, we supplement some by taking some videos from the clean subset and adding random sparse noise onto their feature (raw pixel) matrices to simulate the occluded videos. The random sparse noise is a matrix, 10% elements of which are randomly distributed values larger than 2 while the rest are all zeros. This is sufficiently gross corruptions for the normalized clean feature matrix whose entries are all between 0 and 1.

To test the robustness of different methods to image-specific corruptions (outliers), we choose the ETH-80 dataset for performance evaluation. The image-specific corruptions are generated by randomly replacing 20% columns of the clean feature matrix of each query set with random noise vectors as outliers. The original feature matrix of query set is full of two or three-digit integers while the outliers are anomalous vectors composed of decimals whose amplitudes do not exceed 3. So the outliers are entirely away from the feature space of query set. Then the corrupted query-set matrices are used for testing.

Table 6 gives the recognition results of various methods on two datasets of different kinds of corruptions. It is clear that the proposed robust model achieves the best matching performance under two kinds of gross corruptions, with just a little degradation from the non-corruption case. It can be observed that the proposed method has the 8% and 6% performance improvement on Honda and ETH-80 datasets respectively, compared to the second best method RHISCRC [19]. The success of RHISCRC may come from the $l_{1}$-norm constraints used on both *a* and β[19] which enforce that only a few extremely similar images in query and gallery sets participate in constructing hulls and modelling. Thus the corrupted instances are circumvented. In contrast to the poor performance of RHISCRC on the Traffic dataset mentioned in Section 5.5, we can see that the $l_{1}$-norm used here has both advantages and disadvantages, depending on different situations of data distribution.

**Figure 8:**
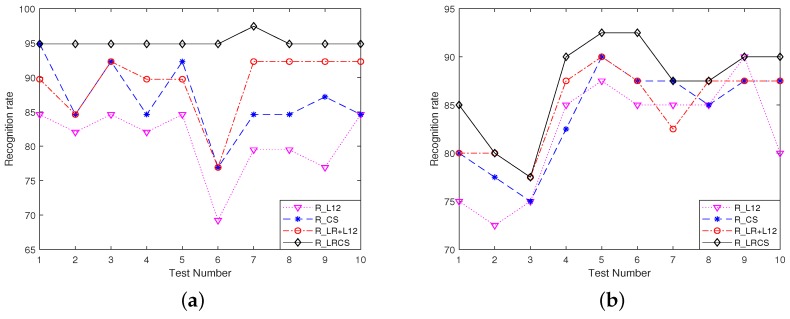
Error terms of two kinds of gross corruptions: (**a**) Random sparse corruptions. (**b**) image-specific corruption.

Furthermore, we also compare the robustness of different joint representation models under the two kinds of corruptions. In order to compare with the proposed R_LRCS fairly, we add the constrained terms standing for corruptions to the other competing joint representation models. For convenience, ‘R_’ abbreviation is added to their names. The recognition performances are illustrated in Figure 8. It can seen that the proposed R_LRCS presents the best recognition rates all the time for two kinds of gross corruptions, indicating the superior robustness of our proposed model. It is also observed that in the majority of the test numbers, the two modified models with low rank regularization added, R_LRCS and R_LR+L12 both perform better than their respective single-constraint models, R_CS and R_L12. The proposed method takes advantage of the competition and collaboration not only within the gallery sets but also considering the inter-instance correlation within the query set. As such, the commonality and variations across the query-set images can be achieved simultaneously through the low rankness and class-level sparsity constraints on the representation coefficients.

## 6. Conclusions

In this paper, we proposed a joint representation model with both low rank and class-level constraints imposed on the representation coefficients to explore fully the inter-instance relationships within the query set for improving image set matching. Compared with joint representation models with other sparsity constraints, the model with class-level sparsity constraint is more appropriate for image set matching, due to its enhanced inter-class discrimination by class label prior. In addition, a low rank regularization is added to the class-level sparse joint representation model, thus both the commonality and variations within the query set are exploited for improving ISM performance. To deal with nonlinear data in real scenarios, the proposed model is then extended to the kernelized version. Furthermore, grossly corrupted data are often encountered in real scenarios, which is ignored by most of the existing ISM methods. For this problem, the proposed model is extended to its robust version to tackle the rand sparse and image-specific corruptions. The optimization problems of the proposed model and its extensions are solved by employing singular value thresholding and block soft thresholding operators to solve different variables in an alternating direction manner. Experiments on five public datasets demonstrate that the proposed method has better performance than the existing joint representation models with various constraints for the ISM problem and also compares favorably with other state-of-the-art methods.

## Figures and Tables

**Table 1:** Some compared methods/different joint representation models vs. their abbreviations.

Abbreviations	Methods
MSM	Mutual Subspace Method [13]
AHISD	Affine Hull based Image Set Distance [18]
CHISD	Convex Hull-based Image Set Distance [18]
SANP	Sparse Approximated Nearest Points [9]
NN_H	Nearest Neighbor classifier based on the Hellinger distance [6]
NN_J	Nearest Neighbor classifier based on the J-divergence [6]
PDL	Prototype Discriminative Learning [8]
RHISCRC	Regularized Hull of Image Set based Collaborative Representation and Classification [19]
RT_LSVM	Reverse Training based Linear Support Vector Machine [4] and GCR [39]
GCR	Group Collaborative Representation [39]
KAHISD	Kernelized AHISD [18]
KCHISD	Kernelized CHISD [18]
KCHISCRC	Kernelized Convex Hull of Image Set based Collaborative Representation and Classification [19]
KRCHISD	Kenrnelized Reduced CHISD [21]
DRM	Deep Reconstruction Models [3]
L1	$l_{1}$-norm regularized general sparse joint representation model
L12	$l_{1,2}$-norm regularized row-level sparse joint representation model
CS	$l_{1,CF}$-norm regularized Class-level Sparse joint representation model
LRCS	Low Rank regularization on Class-level Sparse joint representation model
K_LRCS	Kernelized version of LRCS
LR+L12	Low rank regularization on row-level sparse joint representation model

**Table 2:** Average recognition rates and standard deviations of LRCS with different parameter settings on the Traffic dataset (%).

	Two Parameters	γ		
λ		0.1	0.01	0.001
	0.1	93.71/3.36	91.74/1.93	90.95/1.93
	0.01	93.71/3.1	94.5/2.69	94.88/2.35
	0.001	93.71/3.1	94.89/1.95	**95.28**/**1.26**

**Table 3:** Average recognition rates and standard deviations of K-LRCS with different parameter settings on the Traffic dataset (%).

	Two Parameters	γ		
λ		0.1	0.01	0.001
	0.1	**96.86**/**2.2**	96.47/2.95	96.47/2.95
	0.01	96.47/2.95	96.08/3.71	95.29/4.22
	0.001	96.86/2.2	95.68/2.65	95.68/2.65

**Table 4:** Recognition rates (%) of various joint representation models on Maryland dataset.

Methods	Testing No.										Mean/ Std.
	1	2	3	4	5	6	7	8	9	10	
L1	79.49	82.05	87.18	79.49	76.92	89.74	76.92	79.49	84.62	74.36	81.03/4.87
L12	87.18	89.74	84.62	87.18	84.62	84.62	84.62	82.05	84.62	82.05	85.13/ 2.36
CS	84.62	94.87	89.74	87.18	82.05	94.87	87.18	84.62	89.74	79.49	87.44/ 5.05
LR+L12	82.05	87.18	87.18	82.05	79.49	92.31	79.49	76.92	84.62	79.49	83.08/ 4.71
**LRCS**	97.44	89.74	92.31	92.31	84.62	94.87	79.49	92.31	92.31	82.05	**89.74**/ **5.8**
**K_LRCS**	97.44	89.74	92.31	94.87	82.05	94.87	84.62	92.31	92.31	84.62	**90.51**/ **5.14**

**Table 5:** Average recognition rates and standard deviations (%) of various methods on the five datasets.

	Methods	Honda	YTC	ETH80	Traffic	Maryland
Linear	MSM [13]	90.77/1.32	57.52/5.99	70.25/9.01	88.99/1.72	61.03/5.56
	AHISD [18]	84.62/4.83	57.73/5.42	72.5/8.58	91.76/6.01	69.74/7.43
	CHISD [18]	89.49/ 1.89	60.43/5.95	72/8.8	94.5/1.99	74.62/5.19
	SANP [9]	94.10/3.83	64.61/5.68	73/9.19	92.5/2.43	83.59/6.42
	NN_H [6]	97.18/2.25	54.26/5.08	69.25/5.9	93.32/4.72	82.56/5.77
	NN_J [6]	96.41/2.16	54.68/3.65	75.25/5.46	93.32/4.72	85.38/3.64
	PDL [8]	88.21/2.76	60.64/7.06	75.25/9.16	**95.68**/**2.33**	85.38/5.14
	RT_LSVM [4]	91.54/1.73	48.58/4.71	62.25/6.4	90.94/3.27	9.23/2.76
	RHISCRC [19]	98.46/1.32	59.72/4.61	80.5/8.96	34.28/4.93	83.08/3.86
	GCR [39]	98.21/1.81	64.75/5.12	85.75/7.13	93.37/1.98	85.64/4.15
	**LRCS**	**98.97**/**1.24**	**65.18**/**4.47**	**90.25**/**6.29**	**95.28**/**1.26**	**89.74**/**5.8**
Nonlinear	KAHISD [18]	79.23/4.59	58.16/5.81	73.5/8.51	94.9/3.2	70.26/5.29
	KCHISD [18]	89.49/1.89	60.28/6.33	71/7.09	94.5/1.99	75.13/4.91
	DRM [3]	99.74/0.81	59.15/6.24	65.75/9.21	——	——
	KRCHISD [21]	**100**/**0**	61.21/5.75	81.5/6.63	95.16/2.46	85.13/5.32
	KCHISCRC [19]	**100**/**0**	64.82/4.91	84.25/6.78	95.68/2.33	87.69/4.95
	**K_LRCS**	**100**/**0**	**66.31**/**6.19**	**88.75**/**6.59**	**96.86**/**2.2**	**90.51**/**5.14**

**Table 6:** Recognition rates and standard deviations of various methods on partially occluded Honda and image-specific corrupted ETH-80 datasets.

Methods	Honda	ETH-80
AHISD [18]	8.97/3.87	35.5/3.69
CHISD [18]	68.46/6.51	14.25/3.91
SANP [9]	7.69/1.71	15/5.77
NN_H [6]	72.82/4.22	12.5/0
PDL [8]	35.38/9.03	16/5.55
RT_LSVM [4]	57.95/5.43	12.75/0.79
NN_J [6]	82.05/6.4	78.75/5.68
RHISCRC [19]	87.69/5.1	81/5.92
**R_LRCS**	**95.13**/**0.81**	**87.25**/**5.06**
